# Meta-analysis of associations between telomere length and colorectal cancer survival from observational studies

**DOI:** 10.18632/oncotarget.20055

**Published:** 2017-08-07

**Authors:** Wei Wang, Lei Zheng, Ning Zhou, Na Li, Gilisihan Bulibu, Chunlei Xu, Yi Zhang, Yong Tang

**Affiliations:** ^1^ Department of Digestive Internal Medicine, The Affiliated Tumor Hospital of Xinjiang Medical University, Urumqi, Xin Jiang Province, China; ^2^ Department of Endocrinology, The First Affiliated Hospital of Chinese PLA General Hospital, Beijing, China; ^3^ Department of Pharmacy, The First People's Hospital of Jiashan, Jiashan County, Jiaxing City, Zhejiang Province, China

**Keywords:** colorectal cancer, telomere length, meta-analysis, survival

## Abstract

**Background:**

Telomere length (TL) has been reported to be associated with the risk and survival of several cancers. But it is unclear for the prognostic role of TL in colorectal cancer (CRC).

**Materials and Methods:**

Relevant citations were searched and identified using several major online databases through April 2017 which investigated associations between TL and CRC prognosis. We combined summary estimates using hazard ratios (HRs) with 95% confidence interval (CI), which were pooled using a random-effects model. Overall survival (OS) was set as the primary outcome of interest.

**Results:**

There are 8 cohort studies encompassing 1622 patients included in the meta-analysis. Pooled estimate indicated that long TL was not significantly associated with patient OS (HR 1.26, 95% CI, 0.76 to 2.08). When we conducted subgroup analyses based on baseline charcteristics, we found that long TL (versus short TL) was significantly associated with poor OS in studies conducted in Europe (*n =* 4, HR 2.73, 95% CI, 1.65 to 4.52, I^2^ = 0), using Southern blot to measure TL (*n =* 3, HR 2.93, 95% CI, 1.69 to 5.10, I^2^ = 0) and patients’ age more than 60 years (*n =* 3, HR 2.65, 95% CI, 1.22 to 5.76, I^2^ = 0). We found no significant associations between TL and patient disease-free, recurrence-free or progression-free survival (HR 1.19, 95% CI, 0.45 to 3.15).

**Conclusions:**

Current evidence did not provide solid indication that long TL is significantly associated with improved or poor survival for patients with CRC. Further large sample size prospective cohort studies are warranted to determine the true relationship for specific patients.

## INTRODUCTION

As a critical structure with numerous DNA sequences of TTAGGG tandem repeats located at the end of linear chromosomes in eukaryote cells, telomere has reported to plays an important role in the protection of genomic instability and DNA degradation [1, 2]. The persistent cell proliferation makes telomere shorten year by year, and meanwhile telomerase activity decreases gradually. The chromosome instability resulting from abnormal alteration of telomere length (TL) may lead to carcer occurance [3].

The associations between TL and cancer risk, mortality and progression have been reported in numerous studies with controversial results [4–14]. Svenson et al. found that TL was shorter in tumor tissues than the adjacent non-tumorous tissues [15]. Moreover, studies in a number of malignancies have showed that abnormal alternation of TL in peripheral blood leukocytes is significantly correlated with the mortality and progression of cancer patients, such as breast cancer, prostate cancer, gastric cancer and non-small cell lung cancer [16–19]. the prognostica value of TL for colorectal cancer (CRC) has not been fully determined.

In a meta-analysis conducted by Zhang et al. [20], they found that short telomeres were associated with increased CRC mortality, which was also reported by Jia et al [21]. However, due to the small sample size and great heterogeneity among studies in those two studies, we updated the systematic review and meta-analysis to reevaluate the prognostic significance of TL in CRC.

## RESULTS

### Description of the search and selection of studies

**Figure 1:**
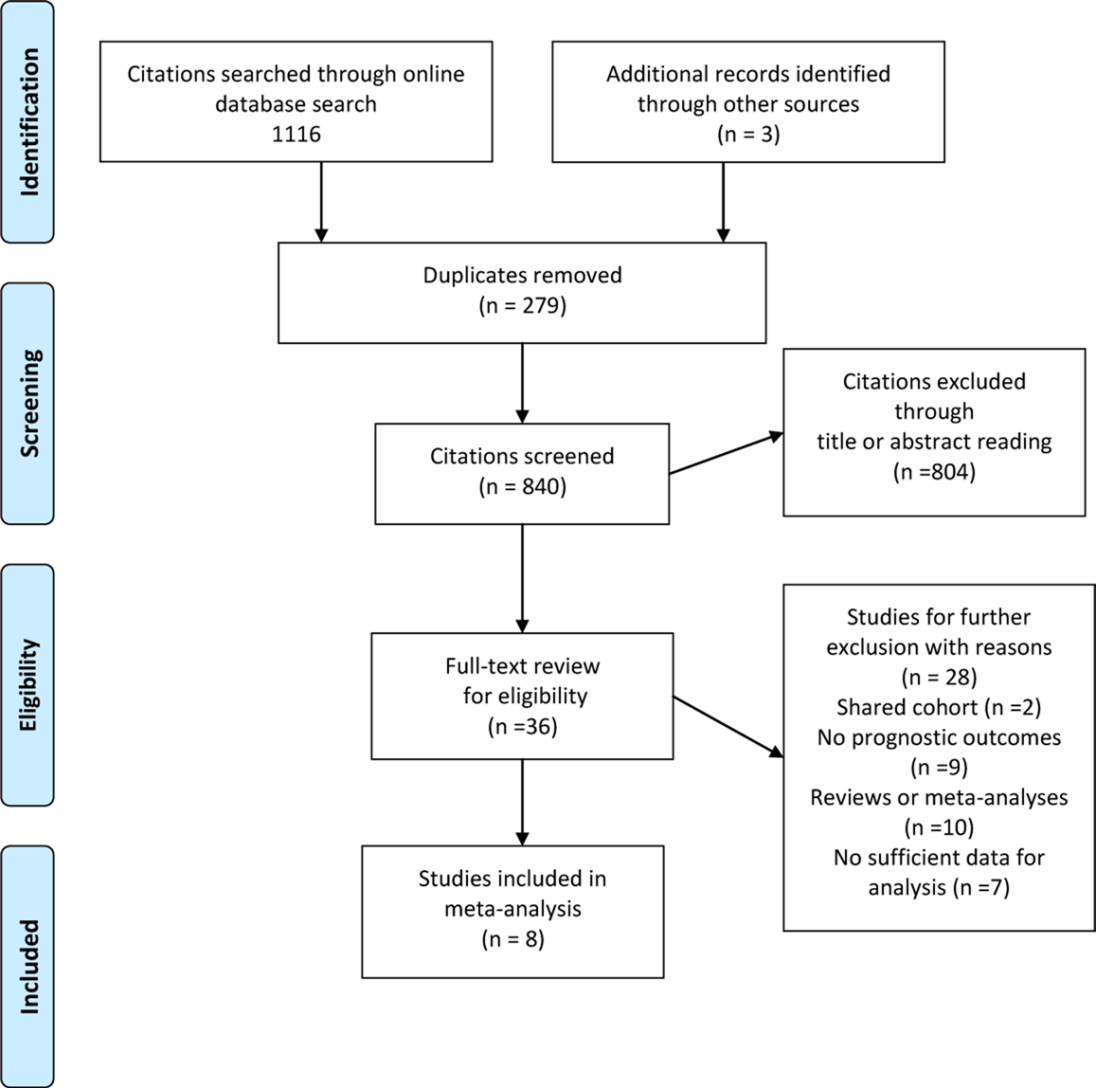
Flow diagram of study selection process investigating effect of telomere length on colorectal cancer prognosis

There were a total of 1119 citations identified for eligibility through database search. After exclusion of duplicate publications, screening of the titles or abstracts, there remained 43 citations for full text review. When excluding the studies unsatisfied for the inclusion criteria, we finally enrolled a total of 8 cohort studies encompassing 1622 patients with a median sample size of 131 (range 57 to 571) for this meta-analysis [22–29]. Data on OS and disease-free survival (DFS)/recurrence-free survival (RFS)/progression-free survival (PFS) were available from 7 and 5 studies, respectively (Figure 1).

### Study characteristics

The basic elements of all the included studies are presented in Table 1. They were all published in English peer-reviewed journals between 2004 and 2016. Five of 8 of the included studies were conducted in Europe, the other three were in USA, Australia and China, respectively. The sample origins were from peripheral leukocytes in two studies [23, 28] while others were from tumor tissues [22, 24–27, 29]. Eight studies investigated I-IV stage CRC patients and only one of them involved stage IV patients [29]. Five studies used qPCR to measure TL while three used Southern blot. The Newcastle-Ottawa scale (NOS) score of methodological quality assessment ranged from 6 to 8 with 5 studies gaining a score of 7 or above (Table 1).

**Table 1:** Study features of included studies investigating the survival outcomes of telomere length for colorectal cancer patients

Author	Country	Sample origin	Sample size	Mean/median age(ys)	Tumor stage	Treatment regimen	Assay/detection method	Cut-off	Follow up period (months)	Outcome reported	Adjustment factors	Quality
Svenson, 2016	Sweden	Peripheral leukocytes	130	Mean 70	I-IV	Surgery	qPCR	NR	Median 202	OS	Age, lymphocyte tumor infiltration, and metastatic status	6
									months.			
Suraweera, 2016	Australia	Tumor tissue	419	Median 70 (30 to 92)	I-IV	Adjuvant treatment	qPCR	NR	Median	OS, DFS	Gender, age, tumor site, stage, differentiation	7
									OS 5.2;			
									PFS 41.6			
Ferandez-Marcelo, 2016	Spain	Tumor tissue	132	Median 71	Dukes Stage A-D	surgery	qPCR	6.35 kb	Median	DFS	Gender, age, tumor location, Dukes stage	7
									60 (1–110).			
Augustine, 2015	USA	Tumor tissue	75	Median 60 (34–93)	IV	EGFR inhibitors treatment	qPCR	Median length	NR	OS, PFS	Age, gender, and race.	6
Chen, 2014	China	Peripheral leukocytes	571	60	I-IV	Surgery+adjuvant chemotherapy	qPCR	0.704	Median	OS, RFS	Age, sex, tumor location	8
									28 (6–60)			
Valls, 2011	Spain	Tumor tissue	147	NR	I-IV	Adjuvant therapy	Southern blot	1	Mean 45	OS	Age,gender, N classification,TNM classification, tumor site, tumor histology, adjuvant therapy	7
Garcia-Aranda, 2006	Spain	Tumor tissue	91	Average 68.60	I-IV	Adjuvant therapy	Southern blot	Mean length	Median	DFS	Gender, age, Dukes stage, tumor location, and differentiation grade of tumors	6
									43.86 (1–77)			
Gertler, 2004	Germany	Tumor tissue	57	Mean 64.6	I-IV	Adjuvant radiochemotherapy	Southern blot	0.9	Median	OS	Tumor site, histologic grade, sex, and age, depth of tumor invasion (pT), lymph node status, and lymphatic invasion and hTERT expression	8
									75.5 (52 to 87)			

Abbreviations: DFS=disease-free survival; hTERT=human telomerase reverse transcriptase; NR=not report; OS=overall survival; PFS=progression-free survival; Qprc = Real-time quantitative PCR; RFS = recurrence-free survival.

### Telomere length and colorectal cancer survival

#### Overall survival

**Figure 2:**
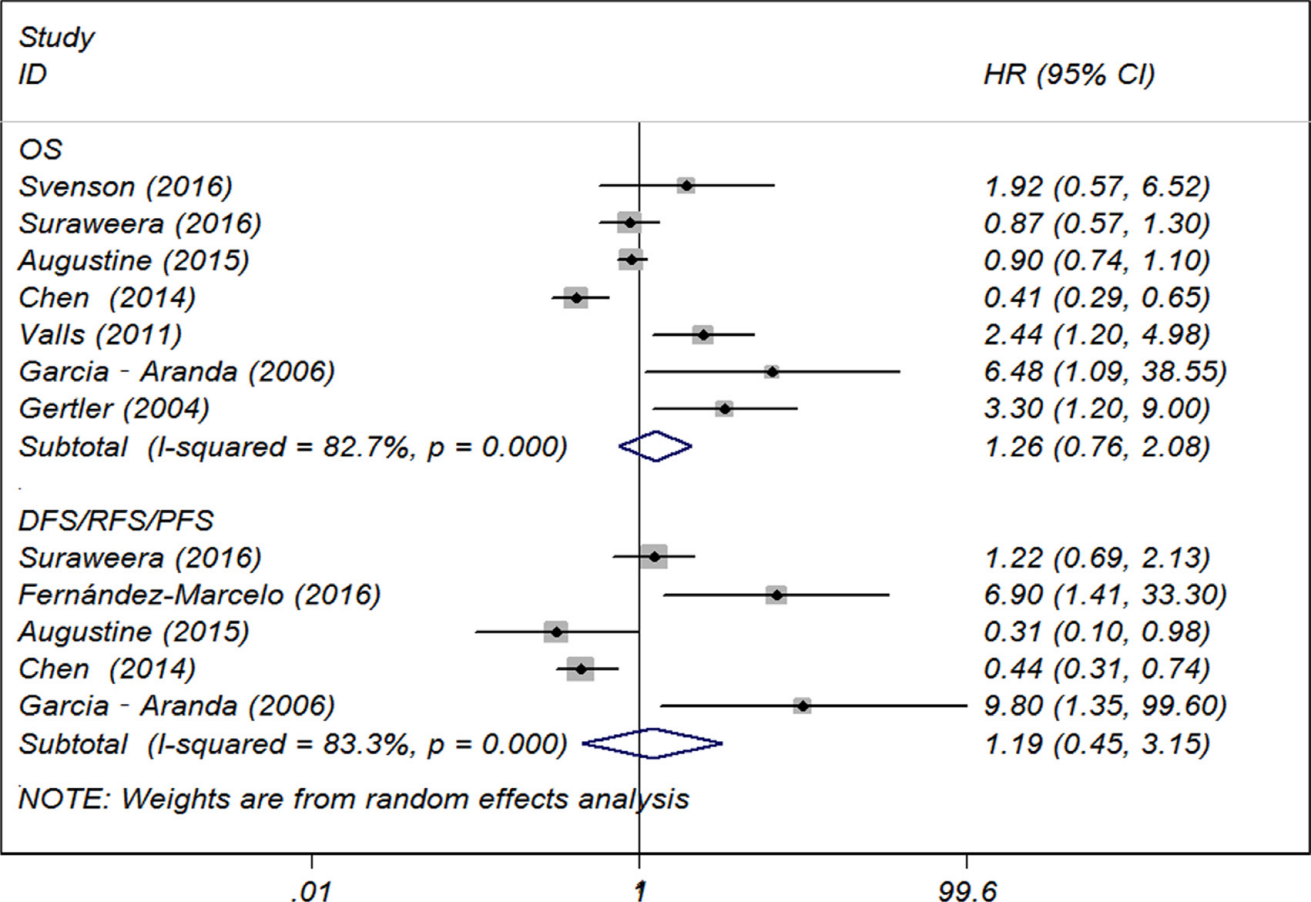
Forest plot of included studies examining the association between telomere length and overall survival

Pooled estimate indicated that long TL was not significantly associated with patient OS (hazard ratio [HR] 1.26, 95% confidence interval [CI], 0.76 to 2.08) (Figure 2) and significant inter-study heterogeneity (I^2^ = 82.7%, *P* < 0.001) was noted. When we conducted subgroup analyses based on baseline charcteristics (Table 2), we found that long TL (versus short TL) was significantly associated with poor OS in studies conducted in Europe (*n* = 4, HR 2.73, 95% CI, 1.65 to 4.52), using Southern blot to measure TL (*n* = 3, HR 2.93, 95% CI, 1.69 to 5.10) and patients’ age more than 60 (*n* = 3, HR 2.65, 95% CI, 1.22 to 5.76). The inter-study heterogeneity of all the three above subgroups decreased significantly (all *I*^*2*^ = 0). However, we did not find significant prognostic associations between TL and CRC OS stratified by sample origin (peripheral blood or tumor tissue), patient mean/median age (< 65 years or ≥ 65 years) or sample size (< 100 or ≥ 100) (Table 2).

**Table 2:** Subgroup analyses based on some baseline features of included studies for overall survival

	Hazard ratio	95% Confidence interval	Degree of heterogeneity (I^2^ statistics; %)	*P*-value	No. of included Studies
**Sample origin**					
**Peripheral blood**	0.74	0.18 to 3.55	82.0	0.76	2
**Tumor tissue**	1.53	0.89 to 2.61	76.7	0.12	5
**Study region**					
**Europe**	2.73	1.65 to 4.52	0	< 0.001	4
**Others**	0.70	0.43 to 1.12	82.7	< 0.001	3
**Mean/median age (ys)**					
**< 65**	0.93	0.42 to 2.03	89.7	0.85	3
**≥ 65**	1.69	0.59 to 4.85	65.1	0.33	3
**Sample size**					
**< 100**	2.19	0.64 to 7.48	81.1	0.21	3
**≥ 100**	1.04	0.47 to 2.30	86.3	0.92	4
**TL detection method**					
**qPCR**	0.77	0.49 to 1.12	78.6	0.26	4
**Southern blot**	2.93	1.69 to 5.10	0	< 0.001	3
**Follow-up period (ms)**					
**< 60**	1.21	0.50 to 2.94	88.1	0.68	4
**≥ 60**	2.65	1.22 to 5.76	0	0.01	3

Abbreviations: ms, months; TL, telomere length; ys, years.

### Disease/recurrence/progression-free survival

We found no significant associations between TL and patient DFS/RFS/PFS (HR 1.19, 95% CI, 0.45 to 3.15) (Figure 2) with significant inter-study heterogeneity (*I*^*2*^ = 83.3%, *P* < 0.001). As limited studies for this outcome subset, we did not further perform subgroup analysis.

### Sensitivity analyses and publication bias

**Figure 3:**
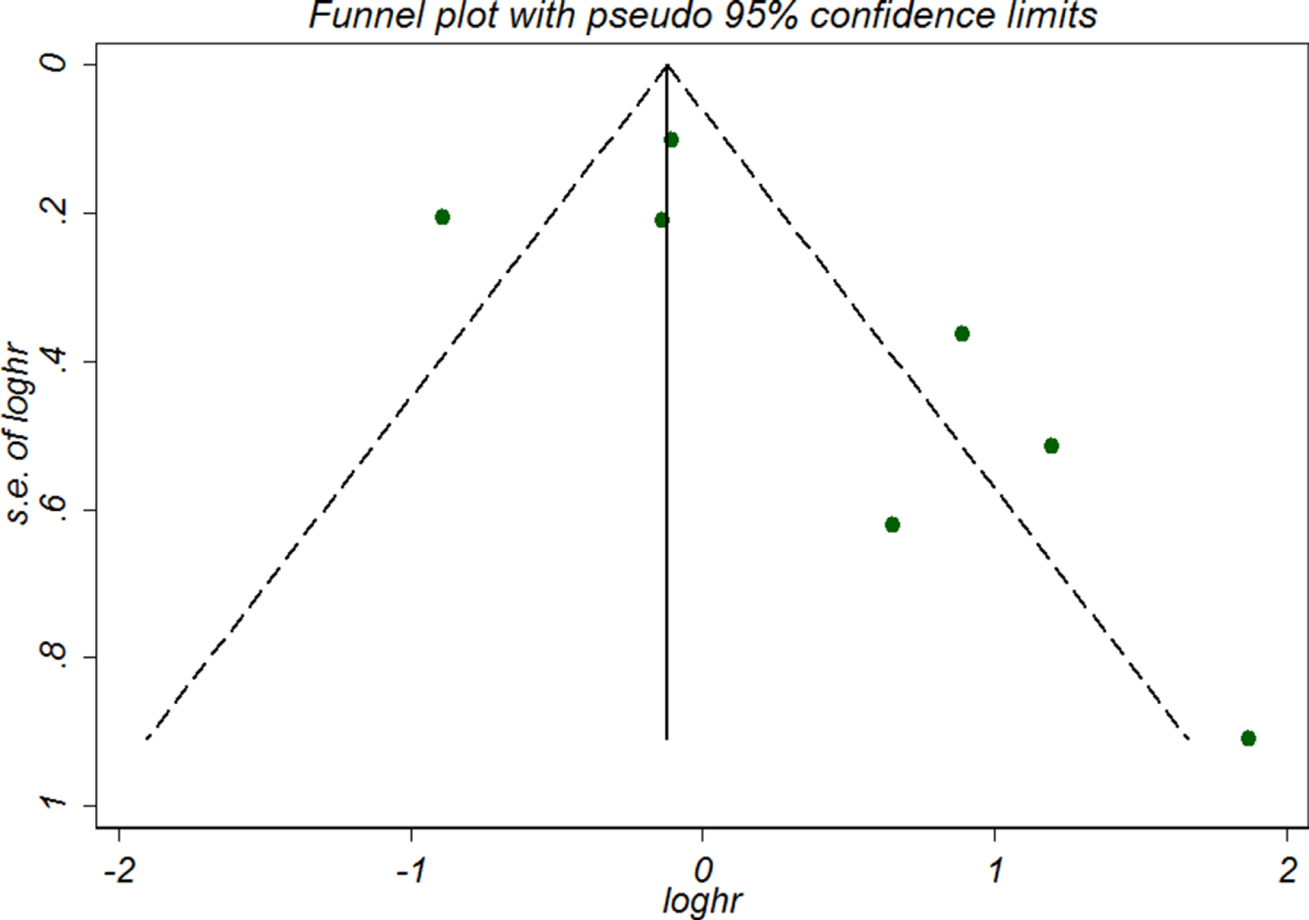
Funnel plot of included studies examining the association between telomere length and overall survival

For OS subgroup, Begg’s test (*P* = 0.368) and Egger’s test (*P* = 0.197) indicated the absence of publication bias (Figure 3). Sensitivity analyses by applying the trim-and-fill method generated similar adjusted estimate (HR 0.98, 95% CI, 0.60 to 1.60) with the primary analyses. As there were only five studies included in the DFS/RFS/PFS subset, the publication bias was not examined.

## DISCUSSION

### Principal findings of this study

The results of this meta-analysis including 8 cohort studies enrolling 1622 patients showed a neutral prognostic effect of TL on CRC survival in terms of OS and DFS/RFS/PFS. Subgroup analyses found that long TL (versus short TL) was significantly associated with poor OS in studies conducted in Europe, using Southern blot to measure TL and patients’ age more than 60 years. We found no significant associations between TL and patient DFS/RFS/PFS.

### Comparisons with previous studies

Although the results reported within the current meta-analysis did not quite agree with previous ones which showed that TL was significantly associated with patient OS. Jia et al. repoted that short TL in peripheral blood leukocytes was significantly associated with poorer OS with fixed-effects model (HR 2.01, 95% CI 1.46 to 2.77) by pooling data from four studies [21]. Zhang et al. also summarized the four studies and obtained similar result [20]. These two studies, however, have limited implications for this topic for the low statistical power with small sample size of included studies. Another recently published study by Adam et.al. [30] also found null association between TL and cancer outcomes. However, it is not just focused on CRC and with limited subgroup analyses.

### Strengths and limitations of the study

There are some limitations to our study. First of all, despite the largest number of studies and patients included on the association between TL and CRC survival, most were small, single-center studies with a higher risk for biases. Secondly, the small number of included studies limited further subgroup analysis and had relatively lower statistical power of the relationship between TL and CRC outcomes. Moreover, DFS, RFS and PFS were combined as the same outcome which could lead to significant heterogeneity as these outcome measures were somewhat different. Moreover, covariants among the included studies are not completely consistent. Finally, several primary selected studies or abstracts were excluded due to the unavailability of original data, which might lead to publication bias for pooled estimates.

There are a number of important strengths for our study. Firstly, we used rigorous search strategies to systematically identify all relevant studies to examine the effect of TL on CRC outcomes. Secondly, significant inter-study heterogeneity was detected in terms of characteristics of observational studies, patient selection (study region, age, tumor stage, etc.), exposure factors (TL measurement methods, cutoffs for long and short TL), therapeutic schedules and outcome reported, though several prespecified subgroup analyses and sensitivity analysis were conducted to explore the sources of heterogeneity. Still several other sources of heterogeneity could not be determined. Additionally, some other factors, such as different study design (prospective versus retrospective), tumor *KRAS/BRAF/PIK3CA* mutation status that affected the survival of CRC patients should also be attributed to some of the heterogeneity. Based on the results of subgroup analyses (Table 2), we noted that I^*2*^ was significantly reduced when analyses were stratified by research region, TL detection method and follow-up period, indicating that these three factors could be part of the sources of heterogeneity. Thirdly, we used a validated scale, NOS to assess the quality of the study evidence for every cohort as was suggested by Cochrane. Fourthly, a relatively large sample size including over 1500 patients made the quantitative analysis more reliable on the association between TL and CRC prognosis. Fifthly, we evaluated each citation by cross-checked means during the process of study screening, data abstraction and quality assessment to maximally minimize selection bias, making the systematic review more objectively.

In summary, current evidence did not provide solid indication that long TL is significantly associated with improved or poor survival for patients with CRC, although the real association remains to be further confirmed by the existing evidence. However, in some subgroups, it provides evidence that in specific population with standard TL detection method (Southern blot) or long-term follow-up (≥ 60 months), long TL may serve as a prognostic biomarker for patients with CRC. Further large sample size prospective cohort studies are warranted to determine the true relationship for some of those specific patients.

## MATERIALS AND METHODS

### Literature search

We developed the search strategies based on the preferred reporting items for systematic reviews and meta-analysis (PRISMA) statement [31]. PubMed, Embase and the Cochrane Library Central Register of Controlled Trials databases from inception through April 2017 were systematically searched for studies investigating the associations between TL and CRC prognosis. Detailed search strategies of the above three databases were presented in Supplementary Table 1. In summary, the following searching terms or phrases with the combinations queries with Boolean logic were used: ‘telomere’ or ‘telomeres’ or ‘telomeric’, ‘colorectal’ or ‘colonic’ or ‘rectal’, ‘cancer’ or ‘neoplas*’ or ‘carcinoma’, ‘mortality’ or ‘prognosis’ or ‘prognostic’ or ‘survival’. We limited the language of included studies only to English, as studies published in other languages were often difficult to interprete and not available for readers.

### Study selection

Two investigators (W.W., L.Z. or N.Z.) evaluated the the titles or abstracts through the primary literature search independently. The final included articles were cross-checked for eligibility by a third investigator (Y.Z. or Y.T.) if necessary until agreement was reached. The inclusion criteria for study eligibility were set as follows: observational (prospective or retrospective) studies reported prognostic outcomes for blood sample or tumor tissue relative TL (long versus short) in CRC patients, and studies provided the definite survival data [risk estimates such as HRs/relative risks (RRs) with 95% CIs obtained directly or estimated through other method [32]. For those overlapped prognostic data, the most recent and comprehensive one was selected for inclusion. Since the low quality of unpublished data and abstracts, we did not include these studies.

### Data extraction

The study elements regarding the study design, patient characteristics, outcomes, exposure of each included study were extracted as follows: the first author and publication year, research country, study sample origin and sample size, mean/median patient age, tumor stage, treatment regimen, assay/detection method for TL, follow up period, outcome reported, adjustment factors and study quality.

### Quality assessment

We applied NOS to assess the quality of each included study, which was calculated based on three major aspects of cohort studies including selection and comparability of study groups, ascertainment of the outcome of interest [33]. Two investigators scored the study quality independently (score range for cohort study 0–9), with a higher score representing better methodologic quality.

### Statistical analysis

All the data were pooled using STATA software 12.0 (StataCorp LP, College Station, TX, USA). We abstracted study estimates (HRs or RRs with 95% CIs) with full adjustments from the included studies and pooled using random-effects model [34] due to the proposed high inter-study heterogeneity. An estimated HR > 1 implied a poor survival for the patient with longer TL (compared with shorter TL). Inter-study heterogeneity was assessed using I^2^ statistic and the Cochrane Q statistic, which was defined as an I^2^ > 50% and *p*-value < 0.10 indicating substantial heterogeneity, respectively [35]. If the potential heterogeneity existed, subgroup analyses were performed based on some of the baseline variables that the heterogeneity could attibute to.

Potential publication bias was evaluated by visual inspection of a funnel plot symmetry and by Egger’s test or Begg’s test [36, 37]. Duval’s nonparametric trim-and-fill method [38] was also applied to adjust the pooled estimates for investigating the potential effect of publication bias.

## SUPPLEMENTARY MATERIALS TABLE


