# Supplementary material for: Meta-analysis of associations between telomere length and colorectal cancer survival from observational studies
Source: Oncotarget. 2017 Aug 7;8(37):62500–7. doi: 10.18632/oncotarget.20055 (PMC5617524; doi:10.18632/oncotarget.20055)
Supplement: Supplementary file 1 [file oncotarget-08-62500-s001.pdf]

Meta-analysis of associations between telomere length and colorectal cancer survival from observational studies

SUPPLEMENTARY MATERIALS

Supplementary Table 1: Search strategy

Table A1. Search strategy for Pubmed
1. "Telomere"[Mesh]
2. (Telomere OR Telomeres OR telomeric)[Title/Abstract]
3. 1 OR 2
4. "Colorectal Neoplasms"[Mesh]
5. "Colonic Neoplasms"[Mesh]
6. "Rectal Neoplasms"[Mesh]
7. (((colorect*[Title/Abstract]) OR colon*[Title/Abstract]) OR rectum[Title/Abstract]) OR rectal[Title/Abstract]
8. ((((((cancer*[Title/Abstract]) OR tumor*[Title/Abstract]) OR tumour*[Title/Abstract]) OR carcinom*[Title/Abstract]) OR neoplas*[Title/Abstract]) OR adenocarcinoma*[Title/Abstract]) OR malignan*[Title/Abstract]
9. 7 AND 8
10. 4 OR 5 OR 6 OR 9
11. 3 AND 10
**Table A2. Search strategy for Embase**
1. 'telomere'/exp
2. (Telomere OR Telomeres OR telomeric):ab,ti
3. 1 OR 2
4. 'colorectal cancer'/exp
5. 'colon cancer'/exp
6. 'rectum cancer'/exp
7. ((colorect* OR colon* OR rectum OR rectal) and (cancer* OR tumor* OR tumour* OR carcinom* OR neoplas* OR adenocarcinoma* OR malignan*)):ab,ti
8. 4 OR 5 OR 6 OR 7
9. 3 AND 8
**Table A3. Search strategy for the Cochrane Library Central Register of Controlled Trials**
1. MeSH descriptor: [Telomere] explode all trees
2. (Telomere or Telomeres or telomeric):ti,ab,kw (Word variations have been searched)
3. 1 OR 2
4. MeSH descriptor: [Colorectal Neoplasms] explode all trees
5. MeSH descriptor: [Colonic Neoplasms] explode all trees
6. MeSH descriptor: [Rectal Neoplasms] explode all trees
7. (colorect* or colon* or rectum or rectal) and (cancer* or tumor* or tumour* or carcinom* or neoplas* or adenocarcinoma* or malignan*):ti,ab,kw
8. 4 OR 5 OR 6 OR 7
9. 3 AND 8

Search included: Pubmed, Embase and the Cochrane Library Central Register of Controlled Trials through April, 2017
